# Supplementary material for: Cumulative environmental exposures adversely impact social behaviour and are associated with dysregulation of genes and proteins involved in epigenetic, ribosomal, and immune regulation in male mice
Source: Inflamm Res. 2026 Jan 8;75(1):17. doi: 10.1007/s00011-025-02152-y (PMC12783315; doi:10.1007/s00011-025-02152-y)
Supplement: Supplementary file 2 — Supplementary Material 2 [file 11_2025_2152_MOESM2_ESM.docx]

**
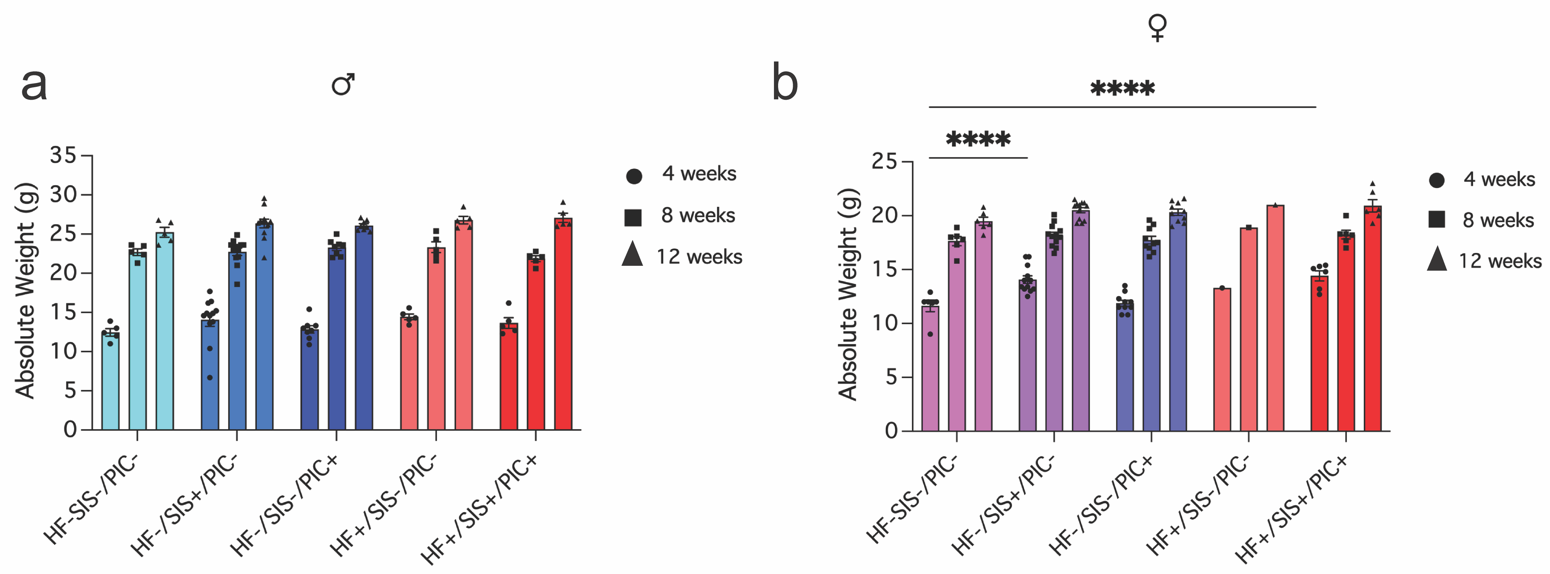
Appendix A:** Supplementary Data

**Figure S1. Offspring demonstrate normal physiological growth into adulthood across stress groups.** (**a**) *males:* Absolute weight (g) across all groups at 4, 8 and 12-weeks of age. Mixed effects model followed by type III ANOVA. Main effect of group (ns); age (****) F2,60 = 1368, p =<0.0001; group x age (*) F8,60 = 2.43, p = 0.023. Followed by Dunnett’s post-hoc test (ns). (**b**) *females:* Absolute weight (g) across all groups at ages described above. Mixed effects model followed by type III ANOVA. Main effect of group (**) F4,30 = 5.2, p = 0.002; age (****) F2,60 = 305.2; group x age (**) F8,60 = 3.34, p = 0.003. Followed by Dunnett’s post-hoc test:4 weeks of age (HF-/SIS-/PIC- vs HF-/SIS+/PIC- and HF+/SIS+/PIC+ (****, p<0.0001)). Data shown as mean ± SEM; mixed effects model. Fixed effects: group, age; random effect: mouse. Legend -males (♂) and females (♀).


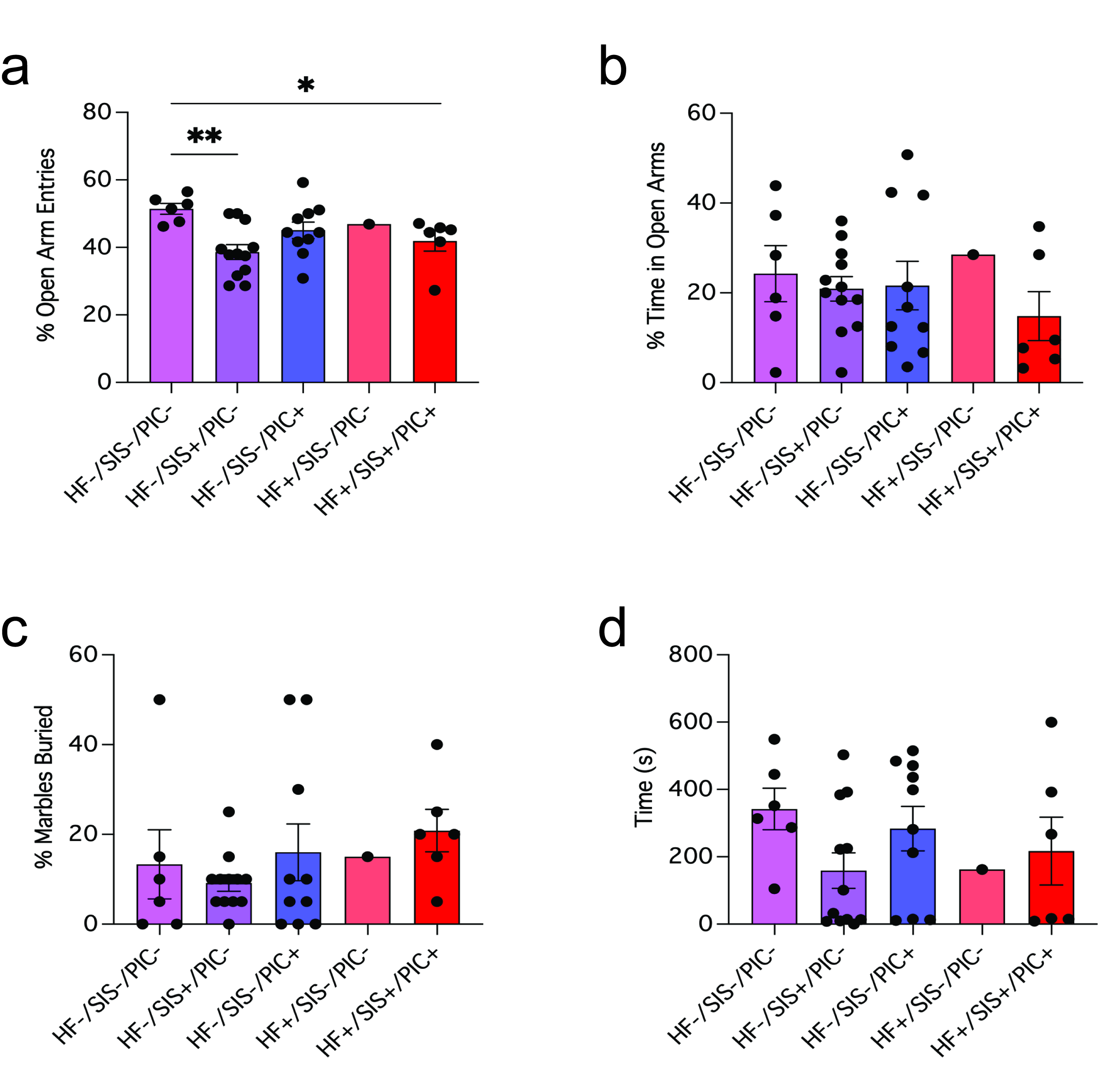


**Figure S2. Female offspring exposed to mSIS alone and all three stress factors display a reduction in open arm entries of the plus maze.** (**a-b**) Elevated plus maze, (**a**) percentage of open arm entries. Ordinary one-way ANOVA. Main effect of group (*) F4,36 = 3.64, p = 0.0137. Followed by Dunnett’s post-hoc test: (HF-/SIS-/PIC- vs HF-/SIS+/PIC- and HF+/SIS+/PIC+ (**, p = 0.0031) and (*, p = 0.035), respectively. (**b**) percentage of time spent in the open arms; (**c**) marble burying test; (**d**) time spent grooming. Data shown as mean ± SEM.

**
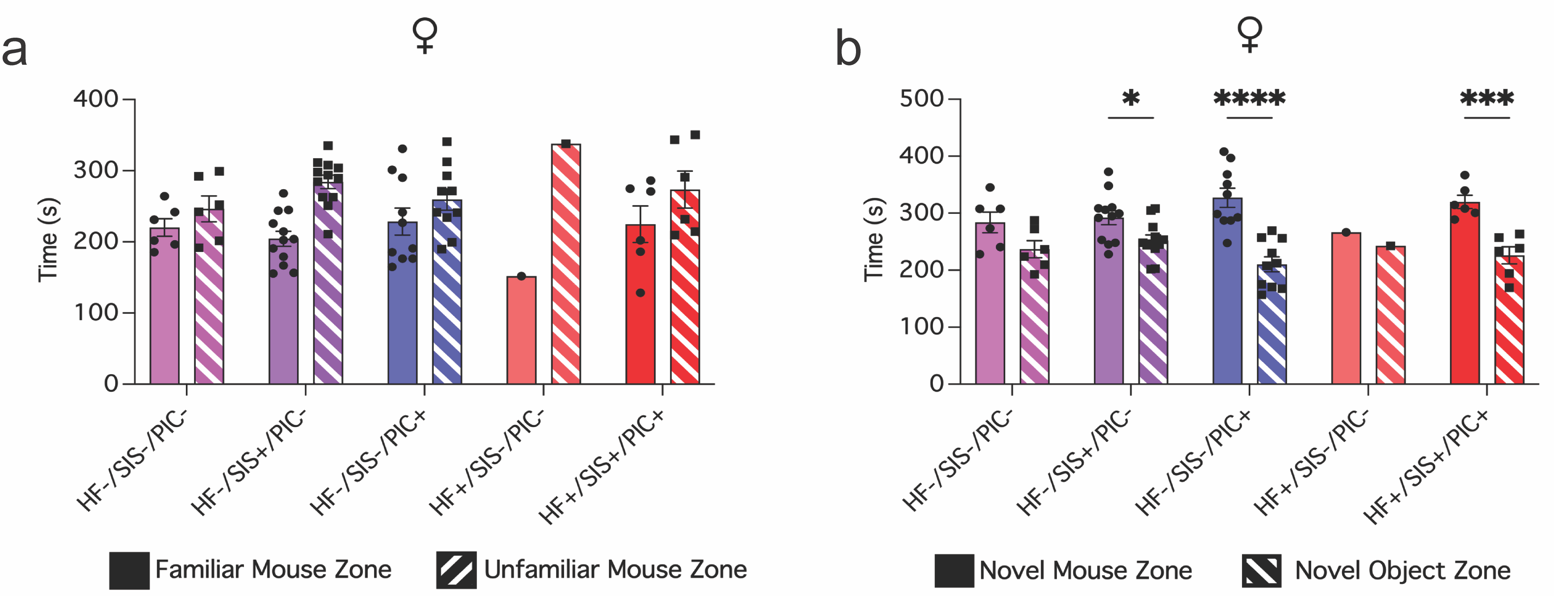
Figure S3. Females across all stress groups do not display social impairments.** (**a-b**) 3-chamber social preference test, (**a**) time (s) spent in familiar mouse zone and unfamiliar mouse zone (**b**) time (s) spent in novel mouse zone and novel object zone. Linear model followed by Type III ANOVA. Main effect of group (ns), zone (ns), group x zone (*) F4,60 = 3.07, p = 0.022. Followed by Tukey’s post-hoc test for within group comparisons between novel mouse zone versus novel object zone: (HF-/SIS-/PIC+ (****,p<0.0001), HF-/SIS+/PIC- (*, p = 0.0201) and HF+/SIS+/PIC+ (***, p = 0.0002). Data shown as mean ± SEM.

**
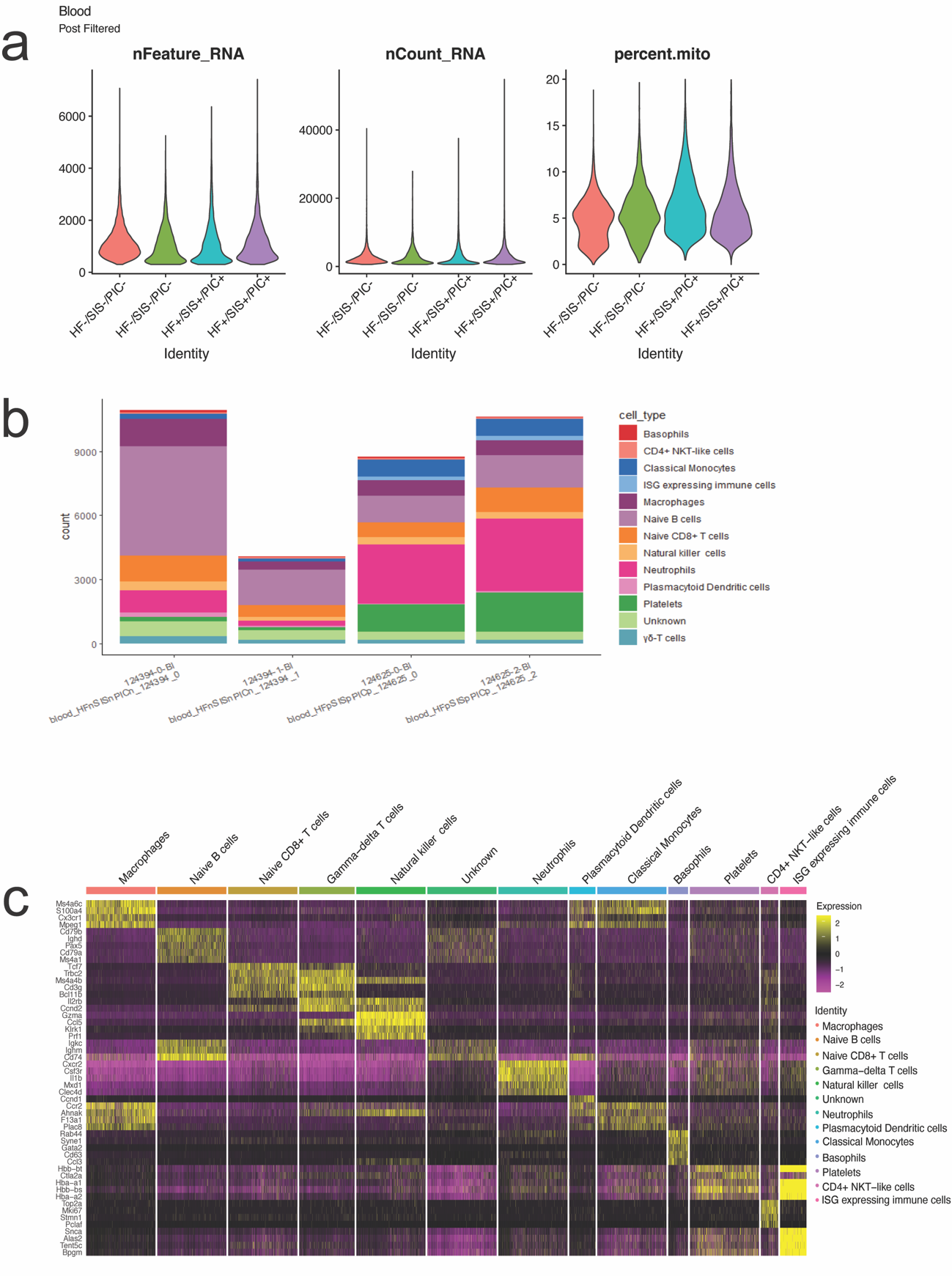
Figure S4.** **scRNA QC summary and heatmap of top differentially expressed genes by cell type for peripheral blood leukocytes.** (**a**) Peripheral blood isolated from male HF+/SIS+/PIC+ and HF-/SIS-/PIC- controls. nFeature_RNA is indicative of the number of genes detected per cell, nCount_RNA is the number of transcripts per cell and percent.mito is indicative of the amount of mitochondrial contamination from dead or dying cells. (**b**) Cell proportions (counts) across samples (not scaled). (**c**) Heatmap displaying top differentially expressed genes per cell type. Data from n = 2 mice / group.

**
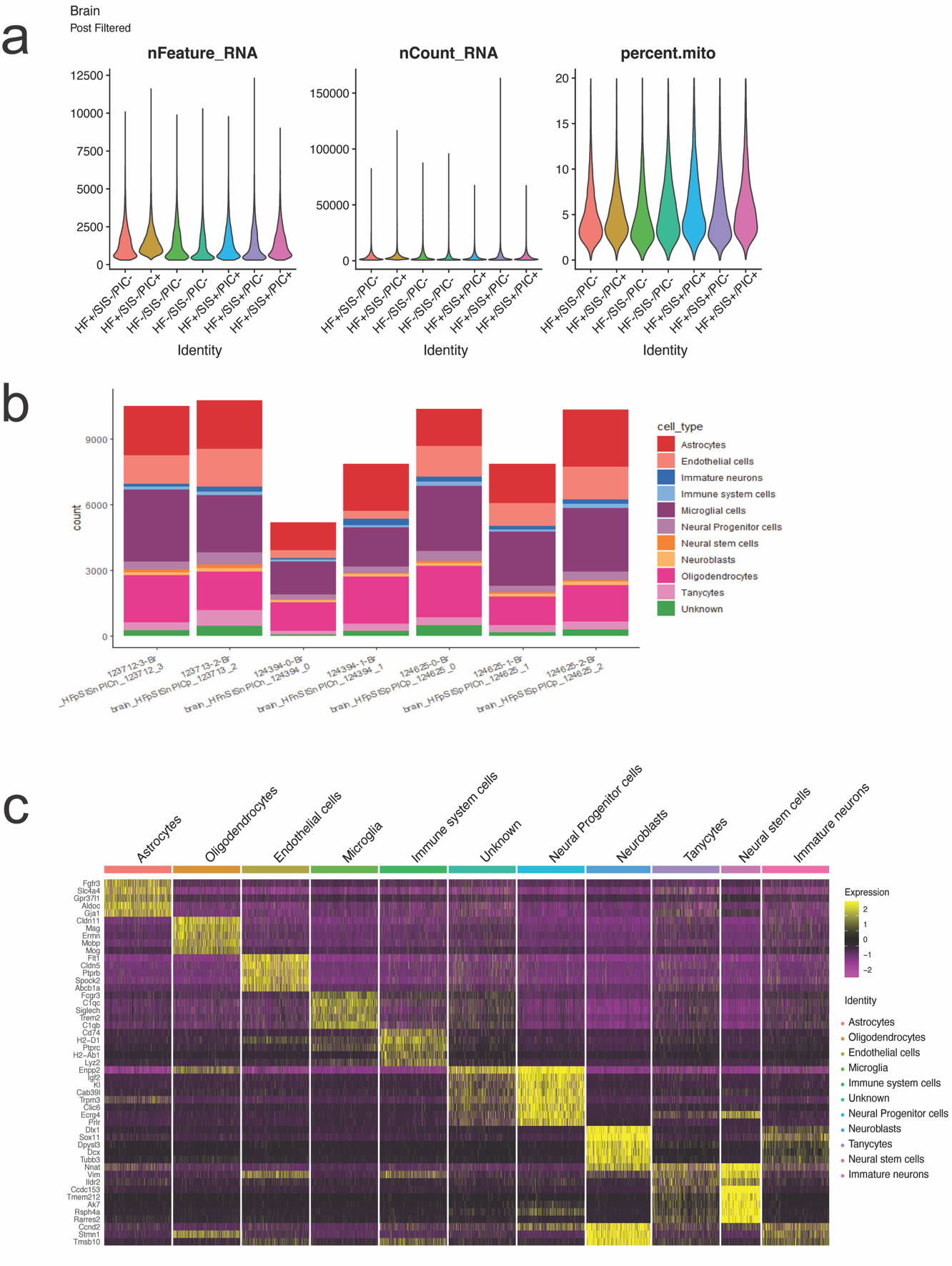
Figure S5**. **scRNA QC summary and heatmap of top differentially expressed genes by cell type for brain cells.** (**a**) Brain cells isolated from HF-/SIS-/PIC-, HF+/SIS+/PIC+ and intermediate male samples that were used for pilot data not described in this manuscript (HF+/SIS-/PIC-, HF+/SIS-/PIC+ and HF+/SIS+/PIC-). nFeature_RNA, nCount_RNA and percent.mito are displayed for number of genes detected per cell, transcripts per cell and proportion of mitochondrial contamination from dead or dying cells. (**b**) Cell proportions across samples (not scaled). (**c**) Heatmap displaying top differentially expressed genes per cell type. Data from n = 2 mice / group between HF+/SIS+/PIC+ and HF-/SIS-/PIC- male controls. Intermediate samples n = 1 / group.


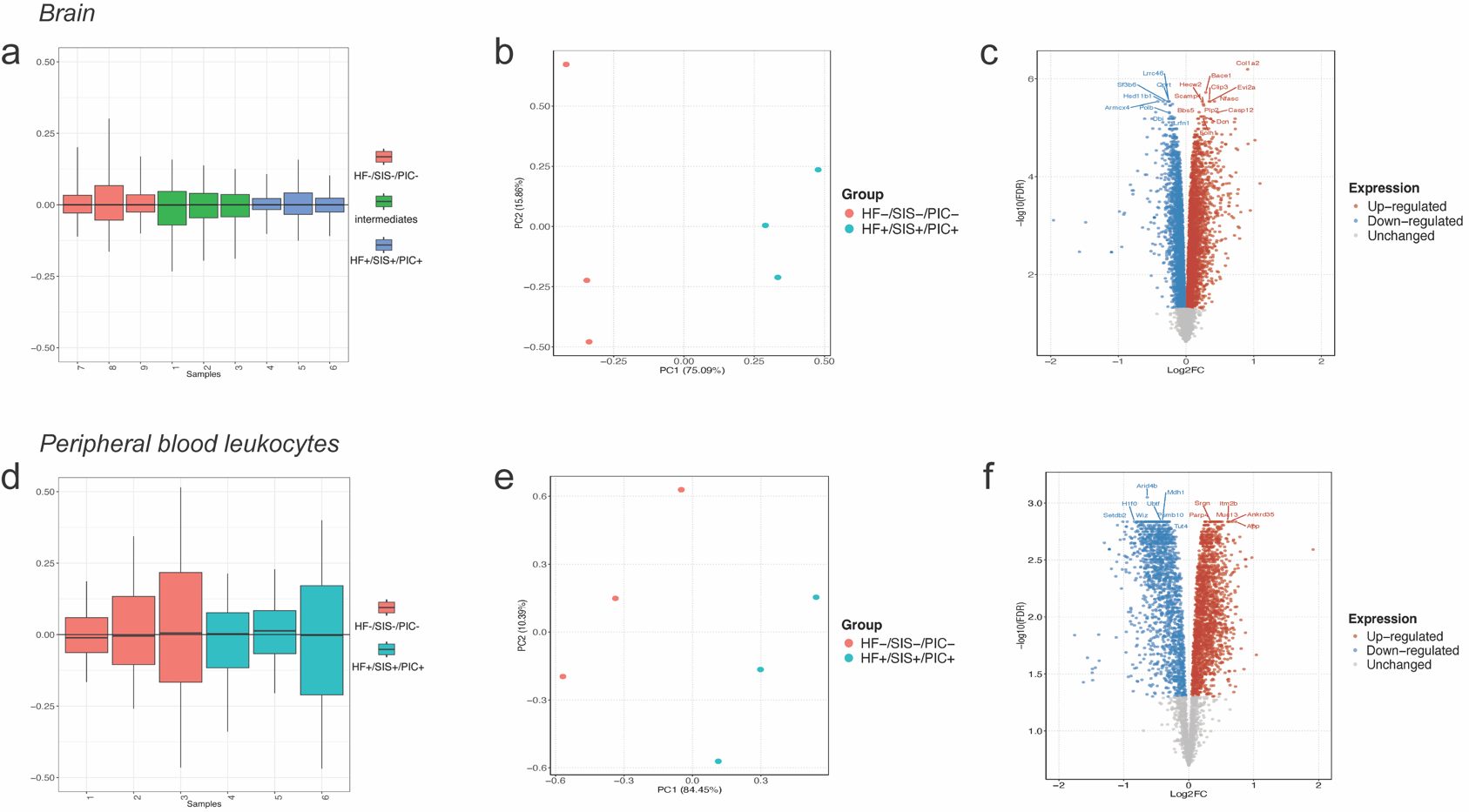


**Figure S6. Proteomics RUV normalisation, principal component analysis (PCA) plots and differentially expressed proteins (DEPs).** (**a**) Box and whiskers relative log expression plot of brain samples after RUV normalisation. Intermediates samples were not described in this manuscript. (**b**) PCA of proteomic data depicting two distinct clusters of stressed (HF+/SIS+/PIC+) versus control (HF-/SIS-/PIC-) offspring in brain tissue. (**c**) Volcano plot depicting top differentially abundant proteins. Coloured dots indicate statistical significance (FDR<0.05). Positive log_2_fold change (Log2FC) (red) illustrates up-regulated expression (e.g. Col1a2, Bace1, Clip3, Evi2a, Nfasc, Casp12, Plp2, Dcn, Folh1, Bbs5, Scamp4, Hecw2), and negative log_2_ fold change (blue) illustrates down-regulated expression (e.g. Lrrc46, Qprt, Lrfn1, Dbi, Polb, Hsd11b1, Sf3b6) in stressed males relative to control males. (**d**) Box and whiskers relative log expression plot of blood samples after RUV normalisation. (**e**) PCA of proteomic data depicting two distinct clusters of stressed (HF+/SIS+/PIC+) versus control (HF-/SIS-/PIC-) offspring in peripheral blood leukocytes. (**f**) Volcano plot depicting top differentially abundant proteins. Coloured dots indicate statistical significance (FDR<0.05). Positive log_2_fold change (Log2FC) (red) illustrates up-regulated protein expression (e.g. Srgn, Itm2b, Muc13, Parp4, App, Ankrd35) and negative log_2_fold change (blue) illustrates down-regulated protein expression (e.g. Arid4b, Mdh1, Psmb10, Tut4, Wiz, H1f0, Setdb2, Ubtf) in stressed males relative to control males. Data from n = 3 mice / group.
